# Efficacy of Antibiotic-Loaded Stimulan Beads Against Biofilms on Clinically Relevant Orthopedic Implant Surfaces

**DOI:** 10.3390/antibiotics15080752

**Published:** 2026-08-04

**Authors:** Tripti Thapa Gupta, Nathan Sacaria, Phillip A. Laycock, Sean S. Aiken, Paul Stoodley, Daniel J. Wozniak

**Affiliations:** 1 Department of Microbial Infection and Immunity, The Ohio State University, Columbus, OH 43210, USA; tripti.gupta@osumc.edu; 2 Department of Microbiology, The Ohio State University, Columbus, OH 43210, USA; sacaria.1@buckeyemail.osu.edu; 3 Biocomposites Ltd., Keele Science Park, Keele, Staffordshire ST5 5NL, UK; 4 Department of Orthopedics, The Ohio State University, Columbus, OH 43210, USA; 5 National Centre for Advanced Tribology at Southampton, Department of Mechanical Engineering, University of Southampton, Southampton SO17 1BJ, UK

**Keywords:** biofilm, periprosthetic joint infections, *Staphylococcus aureus*, calcium sulfate beads, antibiotics

## Abstract

Background: Bacterial biofilms play a key role in causing periprosthetic joint infection (PJI). Current PJI management strategies commonly combine systemic antibiotic therapy with localized delivery such as antibiotic-loaded cement or beads to achieve effective tissue penetration and high antimicrobial concentrations at the implant site. We hypothesized that antibiotic-loaded calcium sulfate beads would effectively eradicate early *Staphylococcus aureus* biofilms but exhibit reduced efficacy against mature biofilms formed in synovial fluid (SF). This study evaluated the efficacy of vancomycin combined with gentamicin (V+G) or tobramycin (V+T) against GFP-expressing *S. aureus* biofilms grown in the presence of SF. Methods: Biofilms were established on clinically relevant orthopedic implant materials such as titanium (Ti), stainless steel (316L), and polyethylene (PE). Early (1-day) and mature (3-day) biofilms were treated with calcium sulfate beads loaded with V+G or V+T. Antimicrobial efficacy was quantified by colony-forming unit (CFU) enumeration. Minimum inhibitory concentration (MIC) testing was performed on surviving populations to assess potential development of antibiotic resistance. Results: Both antibiotic combinations resulted in no detectable viable bacteria in the 1-day biofilm across all tested materials following treatment, demonstrating strong reduction in early biofilm burden below the detection limit. Treatment of mature biofilms resulted in a 4–5 log reduction. MIC analysis of representative bacteria surviving post-treatment indicated no substantial increase in resistance. Conclusions: These findings show that while locally delivered antibiotic combinations eliminate early biofilms, mature biofilms demonstrate significant tolerance. MIC analysis showed little or no change in vancomycin, gentamicin, and tobramycin susceptibility after treatment, indicating no evident increase in planktonic antibiotic resistance among the surviving isolates. Together, these results highlight the importance of early intervention and the need for strategies that disrupt biofilm structure or improve antibiotic penetration to enhance PJI treatment outcomes.

## 1. Introduction

Periprosthetic joint infection (PJI) is a serious and devastating complication following total knee and hip arthroplasty, occurring in approximately 2–2.4% of all joint replacement procedures and representing the most common indication for revision surgery after total knee arthroplasty (TKA) [1,2,3,4,5,6,7]. Despite advances in surgical technique and perioperative care, the incidence of PJI continues to rise, resulting in prolonged hospitalizations, increased patient morbidity, repeated surgical interventions, and rising healthcare costs [8,9,10,11,12]. One of the reasons for increasing PJI cases is the presence of biofilms, groups of microorganisms within a self-produced matrix that are attached to the implant surfaces. Once biofilms are formed, they become tolerant to antibiotic therapy and host immune defenses, leading to the persistence and recurrence of infection despite medical and surgical interventions [13]. Biofilm formation is largely influenced by the surface properties of foreign materials commonly used in orthopedic implant components such as metals and polymers [14,15,16], which provide favorable conditions for bacterial attachment. Among the pathogens associated with PJI, methicillin-resistant *Staphylococcus aureus* (MRSA) poses a significant clinical challenge. MRSA is one of the most prevalent causative organisms in PJI, accounting for approximately 25% of reported cases, and is associated with poor clinical outcomes due to its virulence and limited susceptibility to standard antimicrobial agents [17].

To treat these PJIs, antibiotics such as vancomycin and aminoglycosides like tobramycin or gentamicin are commonly incorporated into polymethylmethacrylate (PMMA) bone cement in the form of spacers and beads [18]. This approach enables the local delivery of high antibiotic concentrations and provides broad-spectrum activity against both Gram-positive and Gram-negative bacteria [19]. Antibiotic-loaded calcium sulfate beads (ALCSBs) have been used as an adjunct to surgical debridement [20]. Conventional systemic antibiotic therapy often fails to eradicate biofilm-associated infections on implant surfaces, as biofilm-embedded bacteria show reduced susceptibility to antimicrobial agents, and incomplete surgical debridement may allow residual bacterial communities to persist [20,21]. Local antibiotic delivery systems were developed to achieve high, sustained antibiotic concentrations directly at the site of infection. ALCSBs represent a biodegradable local antibiotic delivery strategy that enables sustained antibiotic release and does not require surgical removal after implantation [22]. Previous in vitro studies have demonstrated that calcium sulfate beads loaded with antibiotics such as vancomycin and tobramycin can significantly reduce or prevent biofilm formation and planktonic bacterial colonization and show potential in managing biofilm-associated infections [18,23].

Building on this known antimicrobial effect, it is important to consider how these delivery systems may be applied in clinical settings. Orthopedic implants used in joint arthroplasty are composed of various materials such as titanium (Ti) and stainless steel (316L), as well as polymeric components like ultra-high-molecular-weight polyethylene (PE). Biofilm adhesion and antibiotic susceptibility can differ across these surfaces influenced by surface topography, chemistry, and material properties. Understanding how these variables modulate biofilm persistence and response to local antibiotic delivery is critical for improving therapeutic outcomes in PJI. Therefore, the primary goal of this study was to evaluate the efficacy of two clinically relevant antibiotic combinations (vancomycin loaded with gentamicin or tobramycin) delivered via ALCSBs against 1-day and 3-day biofilms formed by a GFP-expressing *S. aureus* strain on different orthopedic surfaces.

## 2. Materials and Methods

### 2.1. Bacterial Strains and Culture Conditions

A green fluorescent protein (GFP) expressing methicillin-resistant *Staphylococcus aureus* (MRSA) strain AH1726 [24] used in this study was provided by Dr. Alexander Horswill at the University of Colorado Anschutz Medical Campus. Strain AH1726 contains a GFP-expressing plasmid in the parental AH1263 background, and both strains form robust biofilms [25]. *S. aureus* (AH1726) was maintained at −80 °C in 20% glycerol and streaked onto tryptic soy agar (TSA) plates, which were incubated overnight at 37 °C in the presence of 5% CO_2_. For each experiment, a single bacterial colony from the streaked plate was used to inoculate 10 mL of tryptic soy broth (TSB). Liquid culture tubes were incubated at 37 °C for 17 to 18 h at 200 rpm.

### 2.2. Biofilm Formation on Coupons in a Well Plate

**Figure 1:**
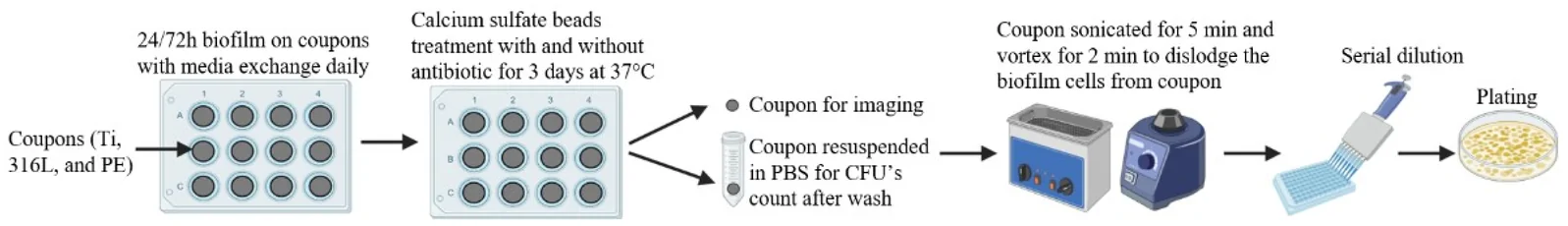
Schematic representation of the experimental methodology used in this study.

Biofilms were formed in 12-well plates containing the respective coupons (Biosurface Technologies, Bozeman, MT, USA) of Ti, 316L, and PE of 12.7 mm diameter and 3.8 mm thickness to simulate clinically relevant orthopedic surfaces (see workflow; Figure 1). Each well was inoculated with 10^6^ CFU/mL of *S. aureus* in 3 mL of TSB supplemented with 10% bovine synovial fluid (SF). SF was incorporated into the growth medium to mimic the in vivo periprosthetic joint environment, as its host proteins (such as fibrinogen and fibronectin) facilitate surface conditioning and support bacterial attachment and biofilm development. The plates were incubated on a rocker at 37 °C to facilitate biofilm formation and ensure uniform bacterial attachment and to closely mimic shear forces encountered in the joint environment. Biofilms were grown for 1 day or 3 days, with the growth medium replaced daily to maintain nutrient availability and remove planktonic cells. After the biofilm formation period, the coupons were washed twice with phosphate-buffered saline (PBS) to remove non-adherent cells and placed in a new well plate containing 4 or 8 antibiotic-loaded calcium sulfate beads per well. Fresh growth medium made in SF was added to each well, and the biofilms were incubated for an additional 3 days on a rocker at 37 °C.

### 2.3. Preparation of Antibiotic-Loaded Calcium Sulfate Beads (ALCSBs)

ALSCBs were prepared using Stimulan Rapid Cure (Biocomposites, Ltd., Keele, UK) 10-cc mixing kits. For each batch, 20 g of calcium sulfate powder was mixed with 1000 mg of vancomycin hydrochloride powder (VWR, Radnor, PA, USA) and either 240 mg of gentamicin sulfate (GOLDBIO, USP Grade, St. Louis, MO, USA) or tobramycin (TCI America^TM^, Portland, OR, USA), depending on the desired antibiotic combination. The antibiotic powders were thoroughly mixed with calcium sulfate powder to ensure a uniform distribution before the addition of 6 mL of sterile liquid provided in the kit. The mixture was stirred continuously for 30 s to form a homogeneous paste. The paste was then pressed into a flexible mold to produce beads with a diameter of 4.8 mm. Beads containing the vancomycin and gentamicin (V+G) combination were allowed to set at RT for 15–20 min, whereas beads containing the vancomycin and tobramycin (V+T) combination required 30–60 min to achieve complete setting. Once set, the beads were ready for experimental use in biofilm treatment assays.

### 2.4. Bacterial Enumeration

**Figure 2:**
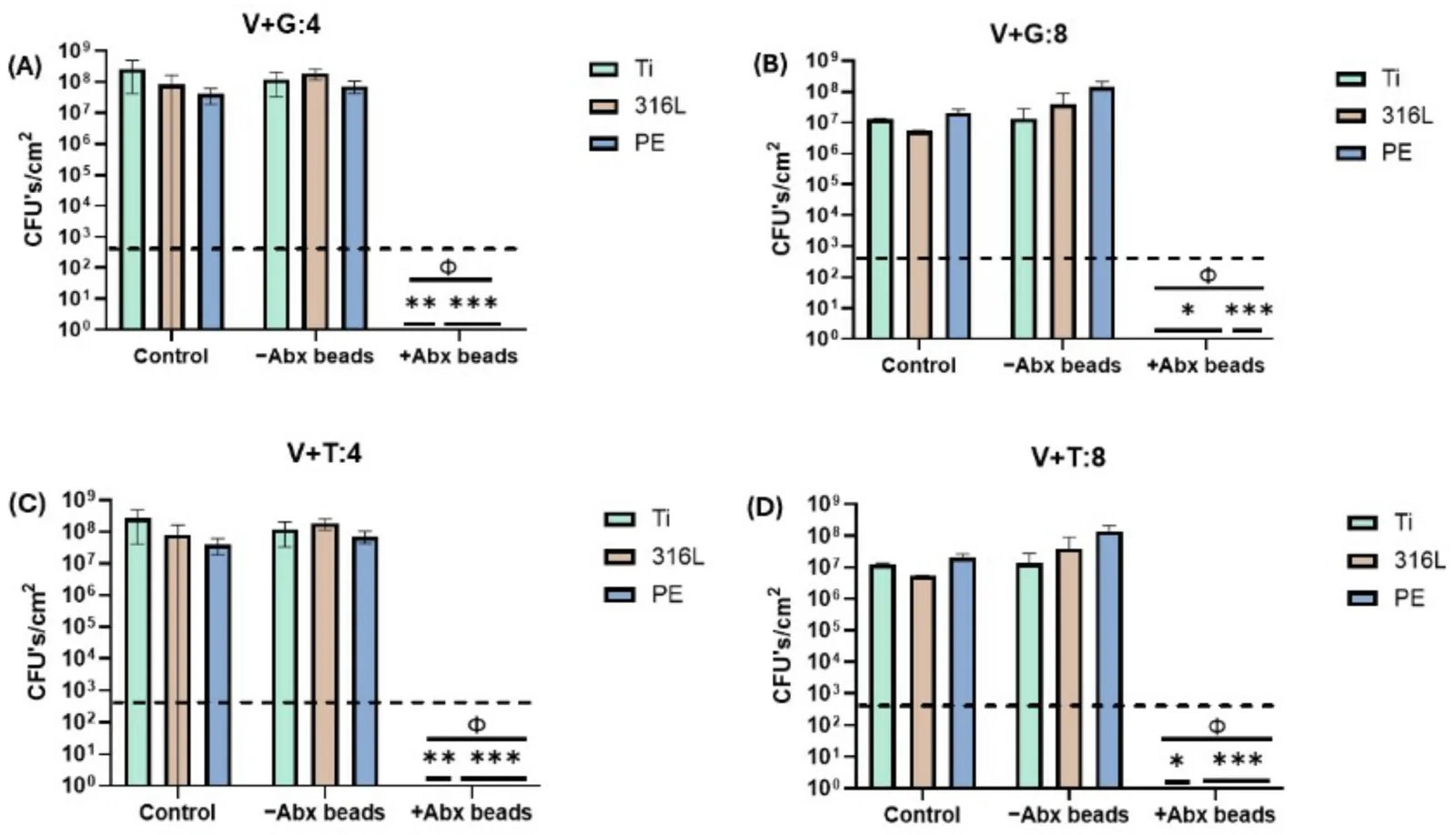
Quantification of viable *S. aureus* following treatment of 1-day biofilms with vancomycin+ gentamicin (V+G) or vancomycin + tobramycin (V+T) delivered via 4 or 8 antibiotic-loaded calcium sulfate beads. Controls are untreated biofilms grown for 1 day without treatment. −Abx beads and +Abx beads denote 1-day biofilms incubated without and with antibiotic-loaded calcium sulfate beads, respectively. The symbol ϕ indicates no detectable CFU. The limit of detection is denoted with a dashed line. Statistical significance was determined via an unpaired two-tailed *t*-test, assuming equal variance and a threshold *p*-value of <0.05. Panels (**A**,**C**): ** *p*-values of 0.0025 (Ti) and 0.0002 (316L); *** *p*-value of 0.0006 (PE). Panels (**B**,**D**): * *p*-values of 0.0105 (Ti) and 0.0194 (316L); *** *p*-value of 0.0008 (PE). Controls shown in panels (**A**,**C**) and (**B**,**D**) were derived from the same experimental control group run under identical conditions and used as a shared reference for comparisons.

**Figure 3:**
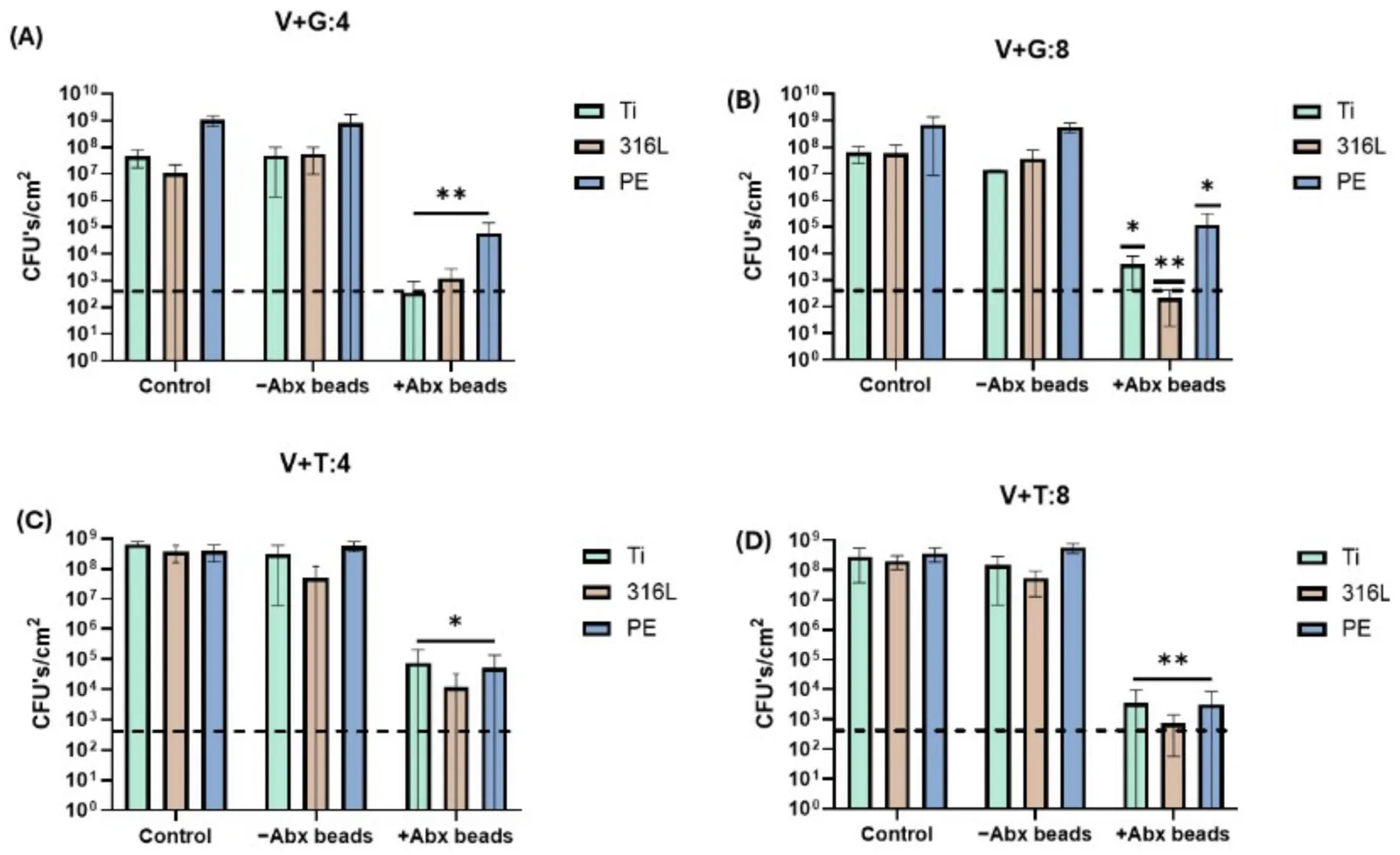
Quantification of viable *S. aureus* following treatment of 3-day biofilms with vancomycin+gentamicin (V+G) or vancomycin+ tobramycin (V+T) delivered via 4 or 8 antibiotic-loaded calcium sulfate beads. Controls are untreated biofilms grown for 3 days without treatment. −Abx beads and +Abx beads denote 3-day biofilms incubated without and with antibiotic-loaded calcium sulfate beads, respectively. The limit of detection is denoted with a dashed line. Statistical significance was determined via an unpaired two-tailed *t*-test, assuming equal variance and a threshold *p*-value of <0.05. Panel (**A**): ** *p*-values of 0.0033 (Ti), 0.0076 (316L), and 0.0023 (PE). Panel (**B**): * *p*-values of 0.0218 (Ti) and 0.0198 (PE), and ** *p*-value of 0.0028 (316L). Panel (**C**): * *p*-values of 0.0293 (Ti), 0.0202 (316L), and 0.0169 (PE). Panel (**D**): ** *p*-values of 0.0093 (Ti), 0.0066 (316L), and 0.0047 (PE).

After the biofilm treatment period, each coupon was gently washed twice with PBS to remove non-adherent planktonic bacteria and then transferred into individual centrifuge tubes containing 5 mL of PBS. To detach biofilm bacteria from the coupon surfaces, the tubes were sonicated for 5 min, followed by vortexing for 2 min to ensure thorough disruption of the biofilm matrix. The resulting bacterial suspensions were subsequently used for quantification by plating with the microdilution method. The bacterial counts obtained in CFU/mL were converted to CFU/cm^2^ after normalizing the surface area of each coupon. This approach allowed for accurate assessment of the biofilm burden on each material surface. The controls in Figure 2 and Figure 3 represent untreated biofilm samples. The 1- and 3-day biofilm controls refer to cells recovered from the surface after 1 and 3 days of biofilm growth without any treatment. −Abx beads in both Figure 2 and Figure 3 denote biofilm samples incubated for 3 days without antibiotic-loaded beads, whereas +Abx beads represent samples incubated for 3 days with antibiotic-loaded calcium sulfate beads.

### 2.5. Confocal Laser Scanning Microscopy Assessment

Following biofilm formation and treatment procedures, the biofilms on the material coupons were visualized using a confocal laser scanning microscope (FluoView FV10i; Olympus, Center Valley, PA, USA) under a 10× and 60× lens objective. Fluorescence emitted by GFP-expressing *S. aureus* allowed for clear identification of treated and untreated biofilm on different surfaces. Due to the working distance limitations of the confocal microscope with the larger coupon (12.7 mm diameter and 3.8 mm thickness) used in the CFU assay, all confocal images presented in this study were acquired using smaller coupons measuring 10 mm in diameter and 2 mm thickness.

### 2.6. Minimum Inhibitory Concentration (MIC) Determination of Surviving Populations Following Antibiotic Bead Exposure

The surviving populations obtained after antibiotic treatment of the 3-day biofilm were subjected to MIC analysis by E-test strip (Liofilchem Diagnostics, Roseto degli Abruzzi, Italy) to determine susceptibility or resistance as described [26]. The post-treatment bacterial suspension was streaked onto TSA plates and incubated overnight to allow colony formation. Following incubation, a single colony was selected and inoculated into TSB media for overnight growth. The overnight culture was diluted to 10^8^ CFU/mL, and 100 µL of the diluted suspension was spread onto Mueller–Hinton agar (MHA) plates using a sterile L-spreader to create a uniform bacterial lawn. E-test strips containing the respective antibiotic were placed on the surface of the bacterial lawn. The plates were incubated at 37 °C for 18–24 h, and the MIC was determined according to the manufacturer’s guidelines after incubation. In parallel, MICs were determined for untreated bacterial populations to serve as controls.

### 2.7. Statistical Analysis

All experiments in this study were performed using two independent biological replicates (*n* = 2), with two technical replicates performed for each biological replicate. Technical replicates were averaged within each biological replicate prior to statistical analysis. CFU data were log_10_-transformed prior to statistical analysis to normalize bacterial count distributions and improve statistical assumptions. Values below the limit of detection (LOD) were considered below the assay detection threshold and were assigned the LOD value prior to log_10_ transformation and statistical analysis. Based on a 10 µL plating volume, 5 mL recovery volume, and a coupon surface area of 1.26 cm^2^, the LOD was calculated as 397 CFU/cm^2^ (log_10_ = 2.6). Statistical analyses were performed using the GraphPad Prism software (version 10). Comparisons were performed between the −Abx beads and +Abx beads groups using an unpaired two-tailed *t*-test assuming equal variance. The threshold for significance was set at a *p*-value of 0.05.

## 3. Results

### 3.1. Vancomycin+Gentamicin (V+G)- and Vancomycin+Tobramycin (V+T)-Loaded Calcium Sulfate Beads Result in No Detectable CFU in Early (1-Day) S. aureus Biofilms

Early biofilms (1-day biofilms) were treated with V+G and V+T using four or eight ALCSBs per coupon. Biofilms were evaluated on Ti, 316L, and PE coupons. There was complete disruption following both antibiotic combination treatments of 1-day biofilm (Figure 2). Controls such as untreated and without antibiotic beads exhibited high bacterial loads across all materials with CFUs in the order of ~10^7^–10^8^ CFU/cm^2^ (Figure 2). Antibiotic-loaded calcium sulfate beads (four and eight) resulted in no detectable CFUs in early *S. aureus* biofilms for both vancomycin–gentamicin and vancomycin–tobramycin groups, with no observed material-dependent differences.

### 3.2. Vancomycin+Gentamicin (V+G)- and Vancomycin+Tobramycin (V+T)-Loaded Calcium Sulfate Beads Decontaminate Mature (3-Day) S. aureus Biofilms

Treatment of 3-day biofilms demonstrated a significant reduction in bacterial burden following exposure to antibiotic-loaded beads. Untreated controls and groups without antibiotic beads maintained high bacterial load (~10^7^–10^9^ CFU/cm^2^; Figure 3). With V+G treatment, four antibiotic-loaded beads led to significant reductions in CFU counts, with a log reduction of 5 on Ti and 316L and 4 on PE when compared with the sample without antibiotic beads. Similarly, on increasing the bead number to eight, there was a log reduction of ~4 on Ti and PE and 5 on 316L.

With V+T treatment, four antibiotic-loaded beads led to a significant reduction in CFU counts, with a log reduction of ~4 on Ti and 316L and 4 on PE when compared with the sample without antibiotic beads. Similarly, on increasing the bead number to eight, there was a log reduction of ~5 on Ti and 316L and 5 on PE. These results indicate reduced efficacy against mature biofilms and material-specific differences in susceptibility.

### 3.3. Confocal Microscopy Shows Effective Reduction in Early (1-Day) Biofilms but Incomplete Disruption of Mature (3-Day) Biofilms Following Antibiotic-Loaded Bead Treatment

**Figure 4:**
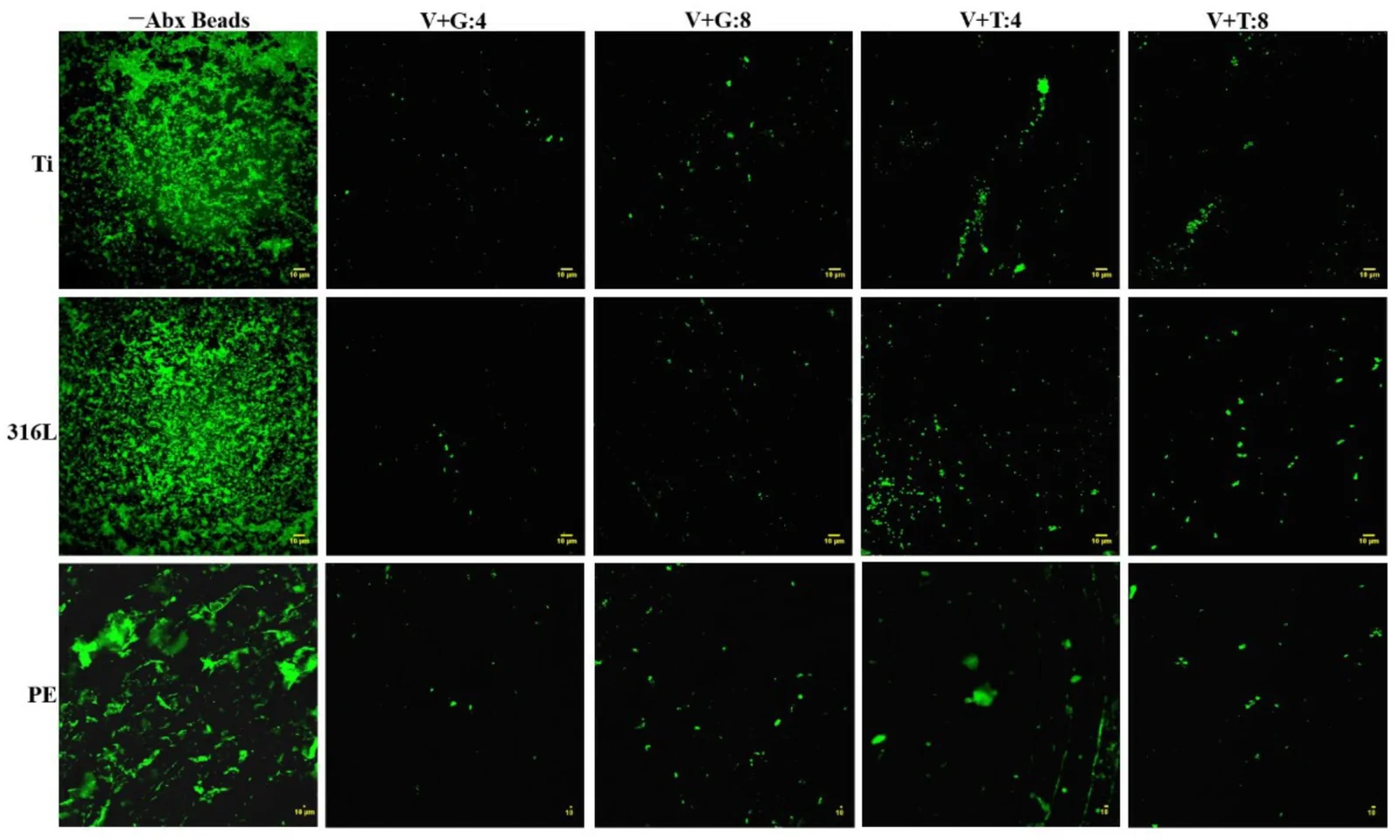
Confocal microscopy images of 1-day *S. aureus* biofilms treated with combination of vancomycin+gentamicin (V+G) and vancomycin+tobramycin (V+T) delivered via 4 or 8 antibiotic-loaded calcium sulfate beads. All images were acquired using a 60× objective, except polyethylene (PE) surfaces, which were imaged at 10× due to microscope focusing limitations. Scale bars = 10 µm.

Confocal microscopy was used to evaluate biofilms before and after treatment on Ti, 316L, and PE (Figure 4).

**Figure 5:**
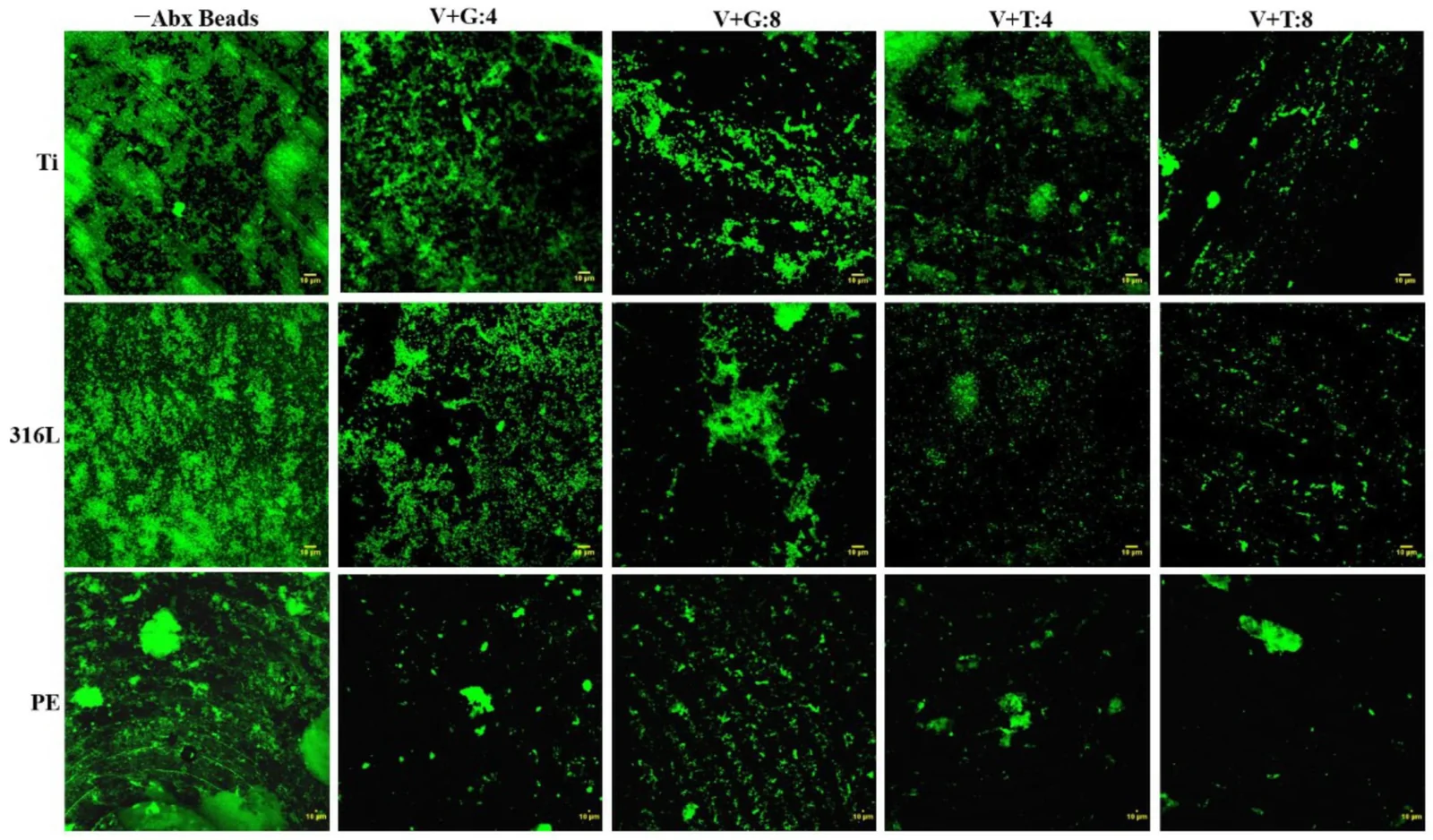
Confocal microscopy images of 3-day *S. aureus* biofilms treated with a combination of vancomycin+gentamicin (V+G) and vancomycin+tobramycin (V+T) delivered via 4 or 8 antibiotic-loaded calcium sulfate beads. All images were acquired using a 60× objective, except polyethylene (PE) surfaces, which were imaged at 10× due to microscope focusing limitations. Scale bars = 10 µm.

On day-1 untreated biofilms, all materials showed substantial biomass with strong green fluorescence on the surface. 316L showed dense and uniform surface coverage, Ti displayed a high but slightly heterogeneous distribution, and PE showed patchy but established early biofilm clusters. The V+G:4 and V+G:8 treatments resulted in substantial reductions in biofilm signals, particularly on Ti and PE. The V+T treatments also reduced biofilm but showed occasional residual fluorescence, especially at the lower dose, suggesting the possible presence of remaining biofilm components on non-culturable bacterial cells. On day 3 (Figure 5), V+G:4 showed reduced biomass, but visible bacterial clusters were observed, particularly on 316L. V+G:8 further provided evidence of decreased biomass, though small aggregates persisted. V+T:4 and V+T:8 showed comparatively less reduction, with persistent fluorescence and bacterial structures across all materials. Overall, confocal imaging demonstrated complete or near-complete eradication of early biofilms, whereas mature biofilms showed only partial, material-dependent disruption following antibiotic-loaded bead treatment.

### 3.4. Minimum Inhibitory Concentration (MIC) of Surviving Populations from Mature Biofilms After Antibiotic-Loaded Bead Exposure Demonstrates Susceptibility

To test whether resistance to V+G or V+T results in emergence of antibiotic-resistant variants, 3-day biofilms on Ti, 316L, and PE surfaces were treated with calcium sulfate beads loaded with V+G or V+T for 3 days. Surviving bacteria were recovered, regrown, and subjected to MIC determination using E-test strips. Without antibiotic beads, the sample showed vancomycin MICs of 2 µg/mL (Ti) and 1.5 µg/mL (316L and PE), while the gentamicin and tobramycin MICs were 0.25 µg/mL (Ti and 316L) and 0.19 µg/mL and 0.25 µg/mL on PE, indicating initial susceptibility (Table 1).

Following V+G and V+T treatment, aminoglycoside (gentamicin and tobramycin) MICs remained constant at 0.25 µg/mL across all surfaces and conditions, indicating no development of resistance or reduced susceptibility. In contrast, in V+G, vancomycin MICs showed modest but measurable variation depending on surface type and bead density. On Ti and 316L, vancomycin MIC did not increase following V+G:4 and V+G:8 treatment while remaining unchanged under the V+G:4 condition. On PE, MICs were more variable after V+G:4 treatment. The MIC did not increase, whereas at eight beads, the MIC remained the same. Overall, all post-treatment MIC values remained within the susceptible range.

## 4. Discussion

Biofilm formation on orthopedic implant surfaces is a major contributor to PJI, making effective strategies for their eradication critically important. In this study, we evaluated the antibiofilm efficacy of vancomycin+gentamicin (V+G)- and vancomycin+tobramycin (V+T)-loaded calcium sulfate beads against 1-day and 3-day in vitro biofilms on clinically relevant orthopedic implant surfaces such as Ti, 316L, and PE. Antibiotic-loaded calcium sulfate beads are used to deliver high local concentrations of antibiotics at infection sites and have been demonstrated to reduce bacterial colonization and biofilm formation in periprosthetic infection models [18,23]. Moreover, these beads can prevent biofilm formation and reduce established in vitro biofilms in multiple bacterial species such as *S. aureus* and *Pseudomonas aeruginosa* [23,26,27].

Our results revealed that early biofilms formed in the presence of SF were highly susceptible to antibiotics loaded with calcium sulfate beads. SF was incorporated into the model to better replicate the physiological environment encountered by bacteria during PJI, where bacteria are exposed to host-derived proteins, nutrients, and immune factors immediately following implantation. Previous studies have demonstrated that SF promotes *S. aureus* aggregation and biofilm-like growth through interactions with host-derived proteins, including fibrinogen and fibronectin, resulting in bacterial phenotypes that more closely resemble those observed in vivo [28,29,30]. Despite the increased physiological complexity of this environment, locally delivered antibiotics remained highly effective against early biofilms. Across all materials, 1-day biofilms treated with V+G or V+T-loaded beads showed substantial reductions in viable cell counts, whereas untreated controls and beads without antibiotics maintained high bacterial burdens. These results indicate that early biofilms are susceptible to local antibiotic concentrations delivered by calcium sulfate beads. Mature biofilms demonstrated higher tolerance and material-dependent variability. While antibiotic-loaded beads still achieved significant reductions, the magnitude of killing was generally lower than in early biofilms, indicating that maturity confers some protective tolerance mechanisms. Interestingly, increasing the number of V+G-loaded beads from four to eight did not consistently improve bacterial killing of 3-day biofilms, particularly on Ti and PE surfaces. This may be because mature biofilms are more difficult for antibiotics to penetrate due to their dense biofilm matrix and the presence of dormant bacterial cells that are less susceptible to antibiotics. In addition, differences in implant material properties may influence biofilm formation and antibiotic penetration, which could explain the material-dependent response. These findings suggest that simply increasing the local antibiotic dose may not always improve the treatment of established biofilms. Consistent with these observations, PE surfaces tend to retain higher CFU counts than Ti or 316L after treatment. The higher bacterial adhesion on PE surfaces is likely due to their increased roughness compared with Ti and 316L, as rougher textures offer greater surface area and depressions that create more favorable sites for bacterial colonization [31]. Both V+G and V+T bead treatments showed similar patterns of efficacy, with both combinations effectively reducing early biofilm loads and achieving moderate reductions in mature biofilms. In this context, the antibiotic combination of V+T used by Knecht et al. [23], showed a 6-log reduction in a 3-day biofilm of *P. aeruginosa* and *S. aureus* on 316L, whereas our study revealed 4-log reductions. While these reductions were substantial, complete eradication was not achieved in 3-day biofilms. Previous studies have demonstrated that rifampicin exhibits enhanced activity against staphylococcal biofilms due to its ability to penetrate the biofilm matrix and target bacteria embedded within the biofilm [27,32]. Therefore, alternative Gram-positive antibiotic regimens or combinations incorporating rifampicin may provide improved CFU reduction in mature biofilms.

Our confocal microscopy analysis revealed that a 1-day biofilm was highly susceptible to both antibiotic combinations, with complete eradication confirmed by CFU analysis. In contrast, 3-day biofilms appeared more tolerant, as evidenced by persistent fluorescence and structured aggregates after treatment. Both V+G and V+T showed almost complete elimination of visible biofilm with some residual fluorescence on the surface. The presence of residual fluorescence signal despite the absence of detectable CFUs in 1-day biofilm suggests that the observed structures likely represent non-viable bacterial cells or residual biofilm matrix, as GFP-tagged bacteria can remain fluorescent even as cultivability declines under stress or starvation conditions [33]. For 3-day biofilms, antibiotic treatment significantly reduced biofilm in all materials, with persistent fluorescence and streak-like bacterial structures across all materials. Overall, imaging suggested incomplete disruption of mature biofilms. However, future studies could incorporate additional viability assays such as live/dead staining or metabolic activity assays to more accurately evaluate the impact of treatment on biofilm integrity.

Furthermore, MIC testing showed that the bacteria remained susceptible to the antibiotics even though the 3-day biofilms were not fully eliminated from the surfaces. Importantly, analysis of MIC fold changes indicated that no true resistance developed under any tested condition, as the aminoglycoside MIC remained constant, while vancomycin showed only slight variation ranging from a 0.5-fold change to no change. These findings indicate that the tested surviving isolates did not exhibit detectable changes in planktonic antibiotic susceptibility following treatment. Importantly, the purpose of the MIC analysis was to evaluate whether surviving bacteria exhibited altered antibiotic susceptibility after exposure to antibiotic-loaded calcium sulfate beads. In this context, the stable MIC values suggest that the recovered populations did not acquire or display measurable resistance following treatment. Any minor variations in the vancomycin MIC likely reflect phenotypic heterogeneity within the biofilm population rather than stable genetic resistance. The observed reductions in some conditions may be due to selective survival of more susceptible cells after treatment, while stable MICs indicate persistence of tolerant subpopulations. Surface properties might also influence, with polyethylene showing greater variability in MIC fold changes compared with titanium and stainless steel. This likely reflects differences in biofilm structure and antibiotic penetration across materials. However, this interpretation is based on MIC testing of representative surviving isolates and, therefore, cannot fully exclude the presence of less-susceptible subpopulations within the overall surviving bacterial populations. Additionally, increasing bead number from four to eight did not consistently improve antimicrobial effects, suggesting that higher antibiotic loading does not necessarily translate into better biofilm clearance and may still allow survival of tolerant cells due to diffusion limitations. These findings have important implications for the management of PJI. The lack of increased MIC values among surviving bacteria suggests that the reduced efficacy of antibiotic-loaded calcium sulfate beads against mature biofilms is primarily associated with biofilm-associated tolerance rather than the development of antibiotic resistance. This observation is consistent with the pathogenesis of chronic PJI, where the biofilm matrix and altered bacterial physiology protect bacteria from antimicrobial therapy despite retaining antibiotic susceptibility. Because orthopedic implant surfaces provide a substrate for bacterial attachment and biofilm maturation, preventing early colonization is critical to limiting persistent infection. Our findings demonstrate that locally delivered antibiotics resulted in no detectable CFUs in early biofilms formed on clinically relevant implant materials but are less effective once mature biofilms become established. This highlights the importance of targeting the initial stages of implant-associated biofilm formation, when bacteria remain more susceptible to antimicrobial therapy. Consequently, strategies that prevent early biofilm establishment or combine local antibiotic delivery with biofilm-disrupting approaches may improve treatment outcomes. Together, our findings highlight the importance of early intervention before mature biofilms become established and support the development of adjunctive therapies to enhance the treatment of orthopedic implant-associated infections.

We acknowledge some limitations of this study. The microscopy data are primarily qualitative and do not include quantitative 3D reconstruction such as biomass, thickness, surface coverage, fluorescence intensity, or aggregate size. Future work will incorporate viability and metabolic staining to differentiate live and dead bacteria on the surfaces. Future studies incorporating viability staining, metabolic activity assays, or regrowth experiments will be required to more definitively assess complete biofilm elimination. In addition, subsequent studies should include in vivo or ex vivo models, a broader range of clinical isolates, direct measurement of local antibiotic release kinetics and concentrations under the experimental conditions to better define antibiotic exposure profiles and their relationship with antibacterial efficacy. Such studies will help determine how bead number, antibiotic release kinetics, and daily medium replacement influence biofilm exposure and treatment outcomes. Furthermore, this study was designed using two antibiotic bead doses (four and eight beads) and two biofilm maturation stages (1-day and 3-day biofilms) to compare treatment efficacy across implant materials. While these conditions enabled evaluation of antibiotic activity against early and mature biofilms, future studies will investigate additional bead doses and intermediate or longer biofilm maturation time points to better characterize dose–response relationships and biofilm maturation dynamics. Additionally, characterization of antibiotic release profiles across different bead doses will provide further insight into the relationship between local antibiotic exposure and biofilm eradication. Additionally, another limitation of this study is that CFU enumeration experiments were performed using two independent biological replicates. Although the results were highly reproducible across experiments and supported by multiple technical replicates, additional biological replicates would further strengthen the statistical robustness and generalizability of the findings. Future studies will include additional independent experiments to further validate these observations.

## 5. Conclusions

This study evaluated vancomycin plus gentamicin (V+G)- and vancomycin plus tobramycin (V+T)-loaded calcium sulfate beads against early and mature *Staphylococcus aureus* biofilms formed on clinically relevant orthopedic implant materials under synovial fluid conditions. We hypothesized that locally delivered antibiotic-loaded calcium sulfate beads would effectively eradicate early biofilms but show reduced efficacy against mature biofilms. As per the hypothesis, the beads resulted in no detectable viable bacteria in 1-day biofilms and achieved 4–5-log reductions in viable bacterial counts in 3-day mature biofilms across all implant materials, although complete elimination of mature biofilms was not observed. MIC analysis of the representative surviving population confirmed that the tested bacteria remained susceptible to the antibiotics, with aminoglycoside MICs remaining unchanged and vancomycin showing only minimal variation, ranging from a 0.5-fold change to no change. A key feature of this study is the use of a synovial fluid-conditioned, multi-implant biofilm model that better replicates the clinical environment of PJI. These findings emphasize the importance of early intervention and suggest that established biofilms may require adjunctive strategies that disrupt biofilm structure or enhance antibiotic penetration. Future studies should extend these findings using in vivo PJI models and explore additional antibiofilm and antibiotic combination strategies to improve treatment of implant-associated infections.

## Figures and Tables

**Table 1:** MICs (μg/mL) of surviving bacterial populations following treatment of 3-day biofilms.

Vancomycin					
Surface	**V+G 4**	**V+G 8**	**V+T 4**	**V+T 8**	**−Abx Beads**
**Ti**	1	1	1	1.5	2
**316L**	1.5	1	1	1.5	1.5
**PE**	0.75	1.5	1	1	1.5
**Gentamicin**					
Surface	**V+G 4**	**V+G 8**			**−Abx Beads**
**Ti**	0.25	0.25			0.25
**316L**	0.25	0.25			0.25
**PE**	0.25	0.25			0.19
**Tobramycin**					
Surface	**V+T 4**	**V+T 8**			**−Abx Beads**
**Ti**	0.25	0.25			0.25
**316L**	0.25	0.25			0.25
**PE**	0.25	0.25			0.25

## Data Availability

The original contributions presented in this study are included in this article. Further inquiries can be directed to the corresponding authors.
